# Effects of dietary n-6:n-3 polyunsaturated fatty acid ratio on growth performance, blood indexes, tissue fatty acid composition and the expression of peroxisome proliferator-activated receptor gamma signaling related genes in finishing pigs

**DOI:** 10.5713/ab.21.0288

**Published:** 2021-10-29

**Authors:** Jing Chen, Hongze Cui, Xianjun Liu, Jiantao Li, Jiaxing Zheng, Xin Li, Liyan Wang

**Affiliations:** 1 College of Animal Science and Veterinary Medicine, Shenyang Agricultural University, Shenyang, Liaoning, 110866, China; 2 Shenyang BoRui Husbandry Technology Company Limited, Xinmin, Liaoning, 110300, China

**Keywords:** Dietary Fatty Acid, Finishing Pig, Gene Expression, Lipid Metabolism, Tissue Fatty Acid Composition

## Abstract

**Objective:**

This study investigated the effects of dietary n-6:n-3 polyunsaturated fatty acid (PUFA) ratio on growth performance, blood indexes, tissue fatty acid composition and the gene expression in finishing pigs.

**Methods:**

Seventy-two crossbred ([Duroc×Landrace]×Yorkshire) barrows (68.5±1.8 kg) were fed one of four isoenergetic and isonitrogenous diets with n-6:n-3 PUFA ratios of 2:1, 3:1, 5:1, and 8:1.

**Results:**

Average daily gain, average daily feed intake and gain-to-feed ratio had quadratic responses but the measurements were increased and then decreased (quadratic, p<0.05). The concentrations of serum triglyceride, total cholesterol and interleukin 6 were linearly increased (p<0.05) with increasing of dietary n-6:n-3 PUFA ratio, while that of high-density lipoprotein cholesterol tended to decrease (p = 0.062), and high-density lipoprotein cholesterol:low-density lipoprotein cholesterol ratio and leptin concentration were linearly decreased (p<0.05). The concentration of serum adiponectin had a quadratic response but the measurement was decreased and then increased (quadratic, p<0.05). The proportion of C18:3n-3 was linearly decreased (p<0.05) in the *longissimus thoracis* (LT) and subcutaneous adipose tissue (SCAT) as dietary n-6:n-3 PUFA ratio increasing, while the proportion of C18:2n-6 and n-6:n-3 PUFA ratio were linearly increased (p<0.05). In addition, the expression levels of peroxisome proliferator-activated receptor gamma (*PPARγ*) and lipoprotein lipase in the LT and SCAT, and adipocyte fatty acid binding protein and hormone-sensitive lipase (*HSL*) in the SCAT had quadratic responses but the measurements were increased and then decreased (quadratic, p<0.05). The expression of *HSL* in the LT was linearly decreased (p<0.05) with increasing of dietary n-6:n-3 PUFA ratio.

**Conclusion:**

Dietary n-6:n-3 PUFA ratio could regulate lipid and fatty acid metabolism in blood and tissue. Reducing dietary n-6:n-3 PUFA ratio (3:1) could appropriately suppress expression of related genes in *PPARγ* signaling, and result in improved growth performance and n-3 PUFA deposition in muscle and adipose tissue in finishing pigs.

## INTRODUCTION

Lipids are key nutrients in the human and animal diet, and their main components are fatty acids. The type and proportion of dietary fatty acids consumed affect not only the nutrition and quality of meat but also health and whole-body physiology functions of humans and animals [1]. Studies have shown that consuming polyunsaturated fatty acids (PUFA), especially n-3 PUFA, can provide more effective protection against inflammation, hepatic steatosis, and cardiovascular diseases [2,3]. However, dietary modification has led to an imbalance in n-6:n-3 PUFA ratio, which would reduce the utilization of n-6 and n-3 PUFA, and even increase the risk of cardiovascular diseases [4,5]. From a biological functional aspect, n-6 and n-3 PUFA share the same enzymes for conversion, and interact with and competitively inhibit each other to synergistically regulate biological processes *in vivo* [6]. It has been demonstrated that a balanced n-6:n-3 PUFA ratio may be more important than n-3 PUFA intake for preventing inflammation-related diseases and other chronic disorders [5]. Therefore, many previous experiments regarding to n-6 and n-3 PUFA for humans and animals have focused on the effects of the n-6:n-3 PUFA ratio rather than the concentration of a particular PUFA [7,8].

Meat is an important source of fat and fatty acids for humans. Although it would be possible to increase the deposition of n-3 PUFA and decrease n-6:n-3 PUFA ratio in the meat by providing n-3 PUFA-rich oils into pig feed, the effects of n-6:n-3 PUFA ratio on growth and lipid metabolism are controversial [7]. Furthermore, few studies have examined the effects of dietary n-6:n-3 PUFA ratio on gene expression patterns of transport, synthesis and catabolism of lipids. In general, dietary fatty acids are incorporated into tissue triglyceride (TG) or converted into their derivative n-6 or n-3 long-chain PUFA, while these lipids are oxidized for energy, or used for synthesis of complex lipids such as membrane phospholipids, or in turn resynthesized into TG. Lipid homeostasis *in vivo* is regulated by transcription factors including peroxisome proliferator-activated receptors-γ (*PPARγ*) [9,10]. Activation of *PPARγ* signaling will stimulate related target genes, such as lipoprotein lipase (*LPL*) [11], adipocyte fatty acid binding protein (*aP2*) [12] and hormone-sensitive lipase (*HSL*) [13], which are responsible for fatty acid transport, fat mobilization and lipolysis, respectively. It is unclear whether changes in dietary n-6:n-3 PUFA ratios affect lipid metabolism through the regulation of related genes in *PPARγ* signaling. Therefore, the objective of the present study was to evaluate the effects of dietary n-6:n-3 PUFA ratio on growth performance, blood lipid and adipocytokine profiles, tissue fatty acid composition and related gene expression in PPARγ signaling in finishing pigs.

## MATERIALS AND METHODS

The experimental procedures involving animals were reviewed and approved by the Institutional Animal Care and Use Committee of College of Animal Science and Veterinary Medicine, Shenyang Agriculture University, China (No. 201906001).

### Experimental animals and diets

A total of 72 crossbred ([Duroc×Landrace]×Yorkshire) barrows with an average initial weight of 68.54±1.80 kg were assigned to four groups in a completely randomized design. Each group consisted of six replicate pens with three pigs per pen. The pigs in the four groups were fed isoenergetic (14.07 MJ mebolizable energy/kg) and isonitrogenous (15.80% protein) diets, prepared using 1.6%, 0.8%, 0.4%, and 0% of linseed oil to replace equivalent amounts of soybean oil so that the dietary n-6:n-3 PUFA ratios of the four diets were about 2:1, 3:1, 5:1, and 8:1, respectively (Tables 1 and 2). The diets were formulated to meet or exceed the NRC [14] nutrient requirements. The oil sources used in the experiment were obtained from Jiuzhou Dadi Biotechnology Co., Ltd. (Liaoning Province, China). The pigs received feed and water *ad libitum* in pens on concrete flooring. The experiment lasted for 45 d.

### Sample collection

At the end of the feeding test, blood samples were obtained via jugular vein puncture from one pig with a representative weight per pen and collected in 10 mL serum blood tubes, and then were centrifuged (1,500×g for 20 min at 4°C) for separation of serum for biochemical analysis. The other pig per pen was randomly chosen and then stunned electrically (240 V, 800 Hz for 5 to 6 s) then exsanguinated and eviscerated. Tissue samples (about 10 g) were collected from the *longissimus thoracis* (LT) and subcutaneous adipose tissue (SCAT) at the level of the last rib from the left-hand side of the carcass. The samples were cut into small pieces, snap-frozen in liquid N_2_ then stored at −80°C for polymerase chain reaction analysis. The other LT and SCAT samples (about 5 g) were excised separately for analysis of their fatty acid composition.

### Growth performance

The body weight and feed intake of the pigs per pen were recorded after an overnight fast at the beginning and the end of the experiment to calculate the average daily gain (ADG), average daily feed intake (ADFI), and gain-to-feed ratio (G:F ratio).

### Serum lipid and adipocytokine analysis

The concentrations of TG, total cholesterol (TC), high-density lipoprotein cholesterol (HDL-C) and low-density lipoprotein cholesterol (LDL-C) were measured by an automatic biochemistry analyzer (Hitachi 747; Hitachi Ltd., Tokyo, Japan) with commercially available kits (Shenyang Dingsheng Trading Co., Ltd., Shenyang, China). The concentrations of leptin (LEP), adiponectin (APN), and interleukin 6 (IL-6) were measured using an automated microplate reader (Model 550; Bio-Rad, Hercules, CA, USA) with enzyme-linked immunosorbent assay kits (Shenyang Jinhui Technology Co., Ltd., Shenyang, China). All the procedures and conditions complied with the instructions provided with the kits.

### Fatty acid composition analysis

Samples of feed and tissues were extracted using a mixture of chloroform and methanol (v/v 2:1) to obtain fatty acid methyl esters using the ISO 5509 method [15]. After phase separation, 2 mL of the upper layer was transferred to a sample injection bottle. The fatty acid composition of the samples was then measured by capillary gas chromatography (Agilent 6890-5973N; Agilent Technologies, Santa Clara, CA, USA). The chromatograph was equipped with a capillary column (30.0 m×0.25 mm i.d. and polyethylene glycol-film, thickness 0.25 μm; Chrompack, Palo Alto, CA, USA) and a flame ionization detector. To optimize separation, the initial oven temperature was set at 80°C, held for 1 min then increased to 220°C at 4°C/min up and held there for 14 min. The carrier gas (helium) flow rate was 0.9 mL/min. Both the injector and detector were set at 230°C and the split ratio was 50:1. The peaks were identified using purified standards and different fatty acids were quantified according the peak area, and expressed as percentage of total fatty acids detected.

### Real-time quantitative polymerase chain reaction analysis

Total RNA was isolated from the LT and SCAT samples using TRIzol Reagent (Sangon Biotech, Co., Ltd., Shanghai, China) according to the manufacturer’s instructions. The quantity and purity of the RNA was determined by optical density (OD)260 and OD280 with their ratio close to 2.0. The specific primers of selected genes, including the housekeeping gene (*β-actin*), *PPARγ*, *aP2*, *LPL*, and *HSL* were designed using Primer Premier 5.0 software (Premier Biosoft, San Francisco, CA, USA) (Table 3). The complementary DNA (cDNA) was generated from 700 ng of DNase-treated RNA. The reverse transcriptase reaction conditions were 25°C for 10 min, 50°C for 30 min then 85°C for 5 min. After reverse transcription, the cDNA was diluted by 6 times using sterilized double-distilled water.

The relative expression levels of the genes were determined using real-time fluorescence quantitative polymerase chain reaction (Roche, Rotkreuz, Switzerland). The cycling conditions were 95°C for 3 min, followed by 45 cycles of 95°C for 5 s and 60°C for 30 s, after which the fluorescence measurements were performed. The comparative C_t_ value method (2^−ΔΔCt^ method) was used to calculate the mRNA expression of genes relative to that of β-actin according to the following formula:


$$2^{-\Delta \Delta \text{Ct}}\;(\Delta \Delta {\text{C}}_{\text{t}}={\left({\text{C}}_{\text{t target gene}}-{\text{C}}_{\text{t }\beta \text{-Actin}}\right)}_{\text{treated}}-{\left({\text{C}}_{\text{t target gene}}-{\text{C}}_{\text{t }\beta \text{-Actin}}\right)}_{\text{untreated}})$$

### Statistical analysis

All data were analyzed by SPSS Statistics 26.0 (IBM Corp., Armonk, NY, USA) and presented as means and the standard errors of the means. Polynomial (linear or quadratic) contrasts (adjusted for the unequal spacing of treatments) were conducted to evaluate the effects of n-6:n-3 PUFA ratios. For growth performance, the pen was used as the experimental unit, while for the serum analysis, tissue fatty acid composition and gene expression the individual pig was used as the experimental unit. A p<0.05 was considered to be statistically significant, whereas 0.05<p<0.10 was considered to have a tendency.

## RESULTS

### Growth performance

As shown in Table 4, the initial weight of pigs was not affected by dietary n-6:n-3 PUFA ratio, while final weight had a quadratic increase (p<0.05) with increasing of dietary n-6:n-3 PUFA ratio. Accordingly, ADG, ADFI, and G:F ratio had quadratic responses but the measurements were rather increased and then decreased (quadratic, p<0.05).

### Serum lipid and adipocytokine profile

The concentrations of serum lipid and adipocytokine are shown in Table 5. The concentrations of TG and TC were linearly increased (p<0.05) with increasing of dietary n-6:n-3 PUFA ratio. The concentration of serum LDL-C was not affected by dietary n-6:n-3 PUFA ratio, while that of HDL-C tended to decrease (p = 0.062) with increasing of dietary n-6:n-3 PUFA ratio, and HDL-C:LDL-C ratio and the concentration of LEP were linearly decreased (p<0.05). However, the concentration of IL-6 was linearly increased (p<0.05) with increasing of dietary n-6:n-3 PUFA ratio. Furthermore, the concentration of APN had a quadratic response, and the measurement was decreased and then increased (quadratic, p<0.05).

### Tissue fatty acid composition

The fatty acid composition in the LT is shown in Table 6. The proportions of C10:0, C14:0, C18:3n-3, and C20:1 were linearly decreased (p<0.05) with increasing of dietary n-6:n-3 PUFA ratio, while that of C18:2n-6 was linearly increased (p<0.05). However, the proportion of C16:1 had a quadratic response, and the measurement was increased and then decreased (quadratic, p<0.05). The proportion of C20:0 also had a quadratic response but the measurement was decreased and then increased (quadratic, p<0.05). Furthermore, n-6:n-3 PUFA ratio in the LT was linearly increased (p<0.05) with increasing of dietary n-6:n-3 PUFA ratio.

The fatty acid composition in the SCAT is shown in Table 7. The proportion of C14:0 had a quadratic response, and the measurement was decreased and then increased (quadratic, p<0.05). The proportion of C18:3n-3 was linearly decreased (p<0.05) with increasing of dietary n-6:n-3 PUFA ratio, and the proportions of C20:1 (p = 0.073) and total saturated fatty acids (SFA) (p = 0.073) tended to decrease. However, the proportion of C18:2n-6 was linearly increased (p<0.05) with increasing of dietary n-6:n-3 PUFA ratio, and that of C20:2n-6 tended to increase (p = 0.086). Moreover, the proportion of C16:1 had a quadratic response, and the measurement was increased and then decreased (quadratic, p<0.05). Consistent with the changes in the LT, n-6:n-3 PUFA ratio in the SCAT was also linearly increased (p<0.05) as dietary n-6:n-3 PUFA ratio increasing.

### Gene expression level

The relative mRNA expression levels of genes in the LT are shown in Table 8. The expression levels of *PPARγ* and *LPL* had quadratic responses, and the measurements were rather increased and then decreased (quadratic, p<0.05). However, the expression level of *HSL* was linearly decreased (p<0.05) with increasing of dietary n-6:n-3 PUFA ratio. Dietary n-6:n-3 PUFA ratios had no effect on the expression level of *aP2*.

The relative mRNA expression levels of genes in the SCAT are shown in Table 9. The expression levels of *PPARγ*, *LPL*, and *HSL* had quadratic responses, and the measurements were increased and then decreased (quadratic, p<0.05). Furthermore, the expression level of *aP2* also had a quadratic response, and the measurement was increased and then decreased (quadratic, p<0.05).

## DISCUSSION

### Effects of dietary n-6:n-3 PUFA ratios on growth performance

Previous studies have shown that fat mixtures with similar metabolizable energy in diet do not affect growth performance and carcass quality of finishing pigs [12,16]. However, in the present study, quadratic increases in ADG, ADFI, and G:F ratio were observed and pigs fed the diets with n-6:n-3 PUFA ratios of 3:1 exhibited higher growth rate and feed efficiency. This means that reducing dietary n-6:n-3 PUFA ratios appropriately tended to grow faster, because these ratios have been reported to increase the deposition of PUFA in tissues and protein synthesis in muscles [7]. In contrast, higher n-6 or n-3 PUFA in diet is highly susceptible to be oxidized preferentially for energy production, therefore decreasing growth performance [5]. This result is in agreement with previous reports showing that diets with n-6:n-3 PUFA ratios of 2.5:1 and 5:1 improved growth performance and health of pigs [8].

### Effects of dietary n-6:n-3 PUFA ratios on serum lipid and adipocytokine profiles

Blood TG and TC levels reflect the nutritional status of animals and are positively associated with adiposity [17], while HDL-C level reduce the risk of coronary artery disease because HDL-C partially inhibits the uptake and degradation of LDL-C, and suppresses LDL-induced changes in sterol level [10]. It has been well demonstrated that n-3 PUFA are beneficial to human health, especially attenuating cardiovascular disease. These protective effects of n-3 PUFA could partly result from their beneficial effects on the lipid profile. As expected, the present study indicated that serum concentrations of TG and TC were decreased and HDL-C:LDL-C ratios were increased with dietary n-6:n-3 PUFA ratios decreasing. These results agreed with the observations of Yang et al [5], who reported that blended oils with lower n-6:n-3 PUFA ratios decreased the serum concentrations of TG, TC and LDL-C and increased the concentration of HDL-C. The hypolipidemic effect of n-3 PUFA is associated with fatty acid oxidation in the liver and skeletal muscle, which may ameliorate dyslipidemia and tissue lipid accumulation. Additionally, n-3 PUFA-mediated reductions in the circulating TG may also attribute to inhibiting hepatic TG synthesis and secretion, reducing the intestinal and hepatic apolipoprotein B-100, which is responsible for LDL production, and increasing LPL activity [2].

Several cytokines such as LEP, APN, and IL-6 are synthesized in adipose tissue. LEP circulates in the body at a concentration highly correlated with the body fat mass and participates in the regulation of food intake and energy expenditure. Our results observed that lower dietary n-6:n-3 PUFA ratio increased the serum concentration of LEP. Similar effects of the n-6:n-3 PUFA ratio on LEP have also been observed in rodents and cultured adipocytes [18]. However, other studies on rats and mice have shown that n-3 PUFA (eicosapentaenoic acid) decreases the production of LEP [19,20]. Differences in the type of n-3 PUFA, dosage and duration may have contributed to differences in outcomes.

It has been demonstrated that APN is an anti-inflammatory and insulin-sensitizing hormone [21]. Studies have demonstrated that the serum concentration of APN is negatively correlated with body mass index, type 2 diabetes and cardiovascular diseases [22]. In the present study, lower n-6:n-3 PUFA ratio promoted the production of APN. Human studies have found that the blood concentration of APN was increased after the consumption of n-3 PUFA regardless of body weight [21]. The n-3 PUFA may influence the concentration of APN directly by interacting with transcription factors, including *PPARγ* or indirectly via unknown mechanisms linked to fatty acid oxidation, synthesis or storage [8]. In addition, the present study showed that lower n-6:n-3 PUFA ratio inhibited the production of IL-6, which agreed with the results reported by Duan et al [8]. It is known that IL-6 is also one of important pro-inflammatory cytokines, which can antagonize anabolic growth factors and thus suppresses growth [7]. Lin et al [23] indicated that n-3 PUFA are anti-inflammatory and decrease the production of these inflammatory cytokines, whereas n-6 PUFA are pro-inflammatory and increase their production. Studies in humans, mice and *in vitro* have indicated that the concentration of IL-6 is decreased with increasing of n-3 PUFA intake [24].

### Effects of dietary n-6:n-3 PUFA ratios on tissue fatty acid composition

Tissue fatty acid plays an important role in the flavour, nutritional value of meat [25]. Previous studies have shown that fatty acid composition of the muscle and fat tissue is affected by dietary fatty acid composition. Decreasing dietary n-6 and elevating n-3 PUFA are highly successful in raising the quantities of n-3 PUFA in pork, thus supplying valuable n-3 PUFA to the human diet [2]. Linseed oil is rich in C18:3n-3, which is usually used as n-3 PUFA source to decrease dietary n-6:n-3 PUFA ratio and improve tissue fatty acid composition. In the present study, as expected, the proportion of C18:3n-3 in the LT and SCAT samples were linearly enhanced as dietary n-3 PUFA level increasing, while the proportion of n-6 PUFA such as C18:2n-6 and C20:2n-6, and n-6:n-3 PUFA ratio were decreased linearly correspondingly. This was in agreement with previous findings that adding oils rich in n-3 PUFA (fish oil or linseed oil) into pig diet could increase the deposition of these fatty acids in the meat [10]. Furthermore, we observed that some of SFA (C10:0, C14:0, and C20:0) and monounsaturated fatty acids (MUFA) (C20:1) were increased in the LT, while C14:0 and C20:1 increased only in the SCAT. We speculate that the deposition of some SFA and MUFA might be associated with specific tissues.

### Effects of dietary n-6:n-3 PUFA ratios on gene expression of tissue

Dietary supplementation with fat or fatty acids can modify the fatty acid composition of tissues by altering the expression of lipid metabolism related genes [8]. Lipid metabolism markers include *PPARγ* signaling and related target genes such as *LPL*, *aP2*, and *HSL*. The *PPARγ* signaling has been identified to facilitate cell enlargement and intracellular TG accumulation [26]. As natural ligand for *PPARγ*, n-3 PUFA can suppress the transcription of lipogenic genes and lipogenesis [8]. As expected, the present study demonstrated that lower n-6:n-3 PUFA ratio significantly attenuated the expression of *PPARγ* and *LPL* compared with higher n-6:n-3 PUFA ratio. Similar studies have been reported by Peng et al [10] and Escobar et al [27], who reported that n-3 PUFA diet decreased the gene expression of *PPARγ* and *LPL*. In contrast, Wang et al [28] reported that *LPL* gene expression was positively associated with the concentration of MUFA such as C16:1 and C18:1, but negatively associated with those of SFA such as C16:0 and C18:0, which indicates that the *LPL* gene may be influenced by more fatty acid types.

It is known that *aP2* is an adipocyte-specific gene secreted from adipocytes and participates in fatty acid uptake and the transport process of intramuscular adipocytes [29]. It has been shown that an elevated serum *aP2* is associated with obesity, insulin resistance, hypertension and cardiovascular disease. Studies in humans and *in vitro* have demonstrated that n-3 PUFA decrease serum *aP2* via reduction in gene expression of *aP2* and *PPARγ* in adipocytes [30]. In the present study, lower n-6:n-3 PUFA ratio decreased expression levels of *aP2* and *PPARγ*. Although these results are similar, there might be a difference between pigs and other animals in regulation of the expression and secretion of *aP2* by dietary n-6:n-3 PUFA ratio. Therefore, further studies of circulating *aP2* and related tissue lipid profiles are necessary for evaluating the effect on n-6:n-3 PUFA ratio on expression and secretion of *aP2*.

In addition, in the present study, the gene expression level of *HSL* was decreased in the LT with increasing of dietary n-6:n-3 PUFA ratio, while the opposite trend was found in the SCAT. It is known that *HSL* is an intracellular enzyme that catalyzes the hydrolysis of TG in adipose tissue and regulates the release of non-esterfied fatty acids from lipid stores. The n-3 PUFA can enhance lipolysis through increasing the expression of HSL and decreasing the expression of *PPARγ* in the LT [7], which may be partly responsible for lower growth performance caused by lower dietary n-6:n-3 PUFA ratio. These results are in agreement with previous reports [2].

Dietary fatty acid composition affects lipid metabolism through many potential mechanisms such as regulation of transcriptional genes and interaction of different signaling pathways [1]. The transcriptional regulation of related genes could be more long-term, and associated with specific tissues and the enrichment of tissues with n-3 and n-6 PUFA. Further research is need to determine the effects of n-6:n-3 PUFA ratio on gene expression involved in lipid metabolism to elucidate the underlying regulation mechanisms.

## CONCLUSION

In conclusion, reducing dietary n-6:n-3 PUFA ratio appropriately exhibited beneficial effects on growth performance, serum lipid and adipocytokine profiles, and modified tissue fatty acid composition to accumulate more n-3 PUFA in tissues. The improvement in lipid metabolism induced by low n-6:n-3 PUFA ratio was associated with suppression of some related genes in *PPARγ* signaling. Further research is necessary to confirm the results and to illustrate the underlying metabolic mechanisms and pathways.

## Figures and Tables

**Table 1:** Composition and analysis of experimental diets (air-dry basis)

Components	n-6:n-3 PUFA ratio			

	2:1	3:1	5:1	8:1
Ingredients (%)				
Corn	67.00	67.00	67.00	67.00
Distillers dried grains with soluble	4.00	4.00	4.00	4.00
Corn germ meal	2.00	2.00	2.00	2.00
Soybean meal (47.5% Protein)	19.00	19.00	19.00	19.00
Wheat bran	3.00	3.00	3.00	3.00
Limestone	1.00	1.00	1.00	1.00
Dicalcium phosphate	0.70	0.70	0.70	0.70
Salt	0.30	0.30	0.30	0.30
Soybean oil^1)^	0.40	1.20	1.60	2.00
Linseed oil	1.60	0.80	0.40	0.00
Premix^2)^	1.00	1.00	1.00	1.00
Chemical composition^3)^				
ME (MJ/kg)	14.07	14.07	14.07	14.07
Crude protein (%)	15.80	15.80	15.80	15.80
Crude lipid (%)	5.04	5.04	5.04	5.04
Crude fiber (%)	3.24	3.24	3.24	3.24
Crude ash (%)	3.94	3.94	3.94	3.94
Lysine (%)	0.88	0.88	0.88	0.88
Methionine + cysteine (%)	0.60	0.60	0.60	0.60
Threonine (%)	0.58	0.58	0.58	0.58

PUFA, polyunsaturated fatty acids; ME, mebolizable energy.

1) To replace equivalent amounts of soybean oil, 1.6%, 0.8%, 0.4%, and 0.0% of linseed oil were used, making the dietary n-6:n-3 PUFA ratios about 2:1, 3:1,5:1 and 8:1, respectively

2) Premix provided per kg diet: vitamin A, 8,000 IU; vitamin B_1_, 15 mg; vitamin B_2_, 30 mg; vitamin B6, 15 mg; vitamin B_12_, 0.5 mg; vitamin D_3_, 3,000 IU; vitamin E, 60 IU; vitamin K, 35 mg; nicotinic acid, 175 mg; pantothenic acid, 50 mg; biotin, 2.5 mg; folic acid, 5 mg; Cu, 3 mg as copper sulfate; Fe, 25 mg as iron sulfate; Zn, 60 mg as zinc sulfate; Mn, 15 mg as magnesium sulfate; I, 0.6 mg as potassium iodate; Se, 0.45 mg as sodium selenate.

3) Calculated values.

**Table 2:** Fatty acid composition of the experimental diets (% total fatty acids)

Fatty acid	n-6:n-3 PUFA ratio			

	2:1	3:1	5:1	8:1
C8:0	0.13	0.09	0.05	0.05
C10:0	0.13	0.09	0.05	0.05
C12:0	1.19	0.93	0.55	0.50
C14:0	0.37	0.39	0.26	0.23
C16:0	12.21	13.54	14.28	14.28
C16:1	0.33	0.27	0.14	0.23
C17:0	0.07	0.08	0.08	0.08
C18:0	3.41	3.81	4.07	3.24
C18:1	23.69	23.68	25.38	25.29
C18:2n-6	39.59	42.35	45.11	48.71
C18:3n-3	17.61	13.35	8.66	6.22
C20:0	0.35	0.42	0.46	0.33
C20:1	0.26	0.27	0.29	0.23
C22:0	0.20	0.28	0.34	0.24
C22:4n-6	0.09	0.10	0.09	0.08
C23:0	0.08	0.13	0.06	0.07
C24:0	0.18	0.21	0.12	0.16
MUFA	24.29	24.22	25.82	25.75
PUFA	57.30	55.80	53.86	55.02
SFA	18.42	19.98	20.33	19.24
n-6:n-3^1)^	2.25	3.18	5.22	7.84

MUFA, monounsaturated fatty acids (the sum of C16:1, C18:1, C20:1); PUFA, polyunsaturated fatty acids (the sum of C18:2n-6, C18:3n-3, C22:4n-6); SFA, saturated fatty acids (the sum of C8:0, C10:0, C12:0, C14:0, C16:0, C17:0, C18:0,C20:0, C22:0, C23:0, C24:0).

1) n-6:n-3 = (C18:2n-6+ C22:4n-6):(C18:3n-3).

**Table 3:** Primers used for real-time polymerase chain reaction

Gene symbol	Primers	Sequences (5′–3′)	Size (bp)
*PPARγ*	Forward	ACAGACCTCAGGCAGATCGT	83
	Reverse	GGGTGAAGGCTCATGTCTGT	
*LPL*	Forward	ACGACGCAGATTTTGTAGACG	124
	Reverse	CCTGGTTGGAAAGTGCCTC	
*aP2*	Forward	ACAGGAAAGTCAAGAGCACCA	126
	Reverse	TGATACATTCCACCACCAACTT	
*HSL*	Forward	CAGGTGTCTTTGCGGGTATT	234
	Reverse	CGAGTTCGGCCAGGTTGT	
*β-actin*	Forward	CCAAAGCCAACCGTGAGA	106
	Reverse	CGGCCAGAGGCGTACAG	

*PPARγ*, peroxisome proliferater-activated receptor gamma; *LPL*, lipoprotein lipase; *aP2*, adipocyte fatty acid binding protein; *HSL*, hormone-sensitive triglyceride lipase.

**Table 4:** Effects of dietary n-6:n-3 PUFA ratios on growth performance of finishing pigs

Items	n-6:n-3 PUFA ratio				SEM	p-value	
	
	2:1	3:1	5:1	8:1		Linear	Quadratic
Initial weight (kg)	68.50	69.44	68.93	68.28	0.48	0.391	0.362
Final weight (kg)	115.41	123.89	121.58	115.94	0.95	0.108	<0.001
ADG (kg/d)	1.04	1.21	1.17	1.06	0.02	0.431	0.001
ADFI (kg/d)	2.86	2.95	2.76	2.68	0.04	0.127	0.002
G:F ratio	0.37	0.41	0.42	0.40	0.01	0.179	<0.001

n = 6.

PUFA, polyunsaturated fatty acids; SEM, standard error of the mean; ADG, average daily gain; ADFI, average daily feed intake; G:F ratio, gain-to-feed ratio.

**Table 5:** Effects of dietary n-6:n-3 PUFA ratios on serum lipid and adipocytokine profiles of finishing pigs

Items	n-6:n-3 PUFA ratio				SEM	p-value	
	
	2:1	3:1	5:1	8:1		Linear	Quadratic
TG (mmol/L)	0.17	0.25	0.30	0.33	0.02	<0.001	0.063
TC (mmol/L)	2.95	3.61	3.64	4.11	0.11	0.027	0.229
HDL-C (mmol/L)	3.84	3.80	3.72	3.69	0.05	0.062	0.156
LDL-C (mmol/L)	1.47	1.54	1.54	1.55	0.03	0.228	0.296
HDL-C:LDL-C ratio	2.61	2.47	2.41	2.38	0.04	0.042	0.069
LEP (μg/L)	0.17	0.14	0.13	0.12	0.47	0.004	0.056
IL-6 (μg/L)	3.43	6.58	6.61	6.62	0.07	0.002	0.095
APN (mg/L)	23.24	21.50	11.07	11.32	0.65	0.024	<0.001

n = 6.

PUFA, polyunsaturated fatty acids; SEM, standard error of the mean; TG, triglyceride; TC, total cholesterol; HDL-C, high-density lipoprotein cholesterol; LDL-C, low-density lipoprotein cholesterol; LEP, leptin; IL-6, interleukin 6; APN, adiponectin.

**Table 6:** Effects of dietary n-6:n-3 PUFA ratios on fatty acid composition of the LT of pigs (% total fatty acids)

Fatty acid	n-6:n-3 PUFA ratio				SEM	p-value	
	
	2:1	3:1	5:1	8:1		Linear	Quadratic
C8:0	0.11	0.10	0.10	0.11	0.01	0.700	0.239
C10:0	0.13	0.12	0.12	0.11	0.01	0.044	0.137
C14:0	1.79	1.54	1.36	1.21	0.04	<0.001	0.349
C16:0	25.55	25.04	25.22	26.34	0.55	0.192	0.218
C16:1	2.55	3.10	2.99	2.07	0.20	0.042	0.002
C17:0	0.16	0.16	0.14	0.15	0.02	0.555	0.684
C18:0	13.44	14.06	13.67	14.07	0.40	0.755	0.890
C18:1	37.65	37.29	36.70	37.06	1.41	0.750	0.884
C18:2n-6	13.42	14.76	15.67	16.14	0.55	0.007	0.017
C18:3n-3	3.06	2.35	2.31	1.21	0.14	<0.001	0.159
C20:0	0.28	0.15	0.18	0.21	0.02	0.384	0.007
C20:1	0.93	0.51	0.60	0.50	0.04	0.003	0.160
C20:2n-6	0.53	0.45	0.50	0.45	0.03	0.191	0.430
C20:4n-6	0.40	0.36	0.44	0.38	0.02	0.935	0.514
MUFA	41.07	40.90	40.29	39.63	1.42	0.672	0.862
PUFA	17.97	17.92	18.92	18.18	0.55	0.613	0.478
SFA	41.46	41.17	40.79	42.19	0.74	0.856	0.133
n-6:n-3^1)^	4.87	6.63	7.19	14.02	0.71	<0.001	0.076

n = 6.

PUFA, polyunsaturated fatty acids; LT, lingissimus thoracis; SEM, standard error of the mean; MUFA, monounsaturated fatty acids (the sum of C16:1, C18:1, C20:1); PUFA, polyunsaturated fatty acids (the sum of C18:2n-6, C18:3n-3, C20:2n-6, C20:4n-6); SFA, saturated fatty acids (the sum of C8:0, C10:0, C14:0, C16:0, C17:0, C18:0, C20:0).

1) n-6:n-3 = (C18:2n-6+C20:2n-6+C20:4n-6):(C18:3n-3).

**Table 7:** Effects of dietary n-6:n-3 PUFA ratios on fatty acid composition of the SCAT of pigs (% total fatty acids)

Fatty acid	n-6:n-3 PUFA ratio				SEM	p-value	
	
	2:1	3:1	5:1	8:1		Linear	Quadratic
C10:0	0.06	0.07	0.06	0.08	0.01	0.165	0.152
C12:0	0.11	0.10	0.09	0.11	0.01	0.887	0.109
C14:0	1.29	1.19	1.15	1.36	0.02	0.075	<0.001
C16:0	23.49	22.65	22.62	23.69	0.82	0.686	0.521
C16:1	1.34	1.44	1.35	1.19	0.04	0.042	<0.001
C17:0	0.20	0.16	0.22	0.20	0.01	0.243	0.426
C18:0	16.04	12.49	14.03	14.03	0.58	0.444	0.170
C18:1	29.46	33.79	31.14	30.66	1.10	0.802	0.472
C18:2n-6	21.56	23.49	24.80	25.26	0.72	0.002	0.134
C18:3n-3	4.34	2.76	2.71	1.78	0.26	<0.001	0.061
C20:0	0.25	0.17	0.21	0.19	0.01	0.138	0.160
C20:1	0.92	0.69	0.58	0.56	0.04	0.073	0.220
C20:2n-6	0.68	0.76	0.78	0.65	0.05	0.446	0.086
C20:3n-6	0.08	0.08	0.08	0.08	0.01	0.911	0.903
C20:4n-6	0.18	0.15	0.16	0.16	0.01	0.474	0.410
MUFA	31.42	35.91	33.07	32.41	1.12	0.583	0.452
PUFA	26.84	28.24	28.53	27.93	0.72	0.452	0.264
SFA	41.44	36.83	38.38	39.66	0.83	0.861	0.073
n-6:n-3^1)^	5.18	6.56	9.53	14.69	1.37	<0.001	0.045

n = 6.

PUFA, polyunsaturated fatty acids; SCAT, subcutaneous adipose tissue; SEM, standard error of the mean; MUFA, monounsaturated fatty acids (the sum of C16:1, C18:1, C20:1); PUFA, polyunsaturated fatty acids (the sum of C18:2n-6, C18:3n-3, C20:2n-6, C20:3n-6, C20:4n-6); SFA, saturated fatty acids (the sum of C10:0, C12:0, C14:0, C16:0, C17:0, C18:0,C20:0).

1) n-6:n-3 = (C18:2n-6+ C20:2n-6+C20:3n-6+ C20:4n-6):(C18:3n-3).

**Table 8:** Effects of dietary n-6:n-3 PUFA ratios on the relative mRNA expression levels of genes in the LT of pigs

Genes	n-6:n-3 PUFA ratio				SEM	p-value	
	
	2:1	3:1	5:1	8:1		Linear	Quadratic
*PPARγ*	1.01	1.81	2.57	1.18	0.11	0.921	<0.001
*LPL*	1.00	0.89	1.52	0.93	0.04	0.810	0.021
*aP2*	0.99	0.95	1.07	0.88	0.05	0.335	0.197
*HSL*	0.97	0.78	0.35	0.28	0.03	0.007	0.012

n = 6.

PUFA, polyunsaturated fatty acids; LT, longissimus thoracis; SEM, standard error of the mean; *PPARγ*, peroxisome proliferator-activated receptor-γ; *LPL*, lipoprotein lipase; *aP2*, adipocyte fatty acid binding protein; *HSL*, hormone-sensitive lipase.

**Table 9:** Effects of dietary n-6:n-3 PUFA ratios on the relative mRNA expression levels of genes in the SCAT of pigs

Genes	n-6:n-3 PUFA ratio				SEM	p-value	
	
	2:1	3:1	5:1	8:1		Linear	Quadratic
*PPARγ*	1.00	1.19	1.90	1.43	0.13	0.127	0.005
*LPL*	1.00	1.80	2.66	1.91	0.06	0.084	<0.001
*aP2*	1.00	1.67	0.83	0.72	0.12	0.069	0.037
*HSL*	0.99	1.63	1.97	1.25	0.05	0.690	<0.001

n = 6.

PUFA, polyunsaturated fatty acids; SCAT, subcutaneous adipose tissue; SEM, standard error of the mean; *PPARγ*, peroxisome proliferator-activated receptor-γ; *LPL*, lipoprotein lipase; *aP2*, adipocyte fatty acid binding protein; *HSL*, hormone-sensitive lipase.
